# Proteomics and Mass Spectrometry for Cancer Biomarker Discovery

**Published:** 2007-10-03

**Authors:** Ming Lu, Kym F. Faull, Julian P. Whitelegge, Jianbo He, Dejun Shen, Romaine E. Saxton, Helena R. Chang

**Affiliations:** 1 Gonda/UCLA Breast Cancer Research Laboratory; 2 Revlon/UCLA Breast Center, Department of Surgery/Oncology, David Geffen School of Medicine, Los Angeles, California; 3 The Pasarow Mass Spectrometry Laboratory, Department of Psychiatry & Biobehavioral and the Neuropsychiatric Semel Institute for Neuroscience and Human Behavior, University of California, Los Angeles; 4 Division of Surgical Oncology, Department of Surgery, David Geffen School of Medicine, Los Angeles, California

**Keywords:** Proteomics, Mass spectrometry, Cancer, Biomarkers

## Abstract

Proteomics is a rapidly advancing field not only in the field of biology but also in translational cancer research. In recent years, mass spectrometry and associated technologies have been explored to identify proteins or a set of proteins specific to a given disease, for the purpose of disease detection and diagnosis. Such biomarkers are being investigated in samples including cells, tissues, serum/plasma, and other types of body fluids. When sufficiently refined, proteomic technologies may pave the way for early detection of cancer or individualized therapy for cancer. Mass spectrometry approaches coupled with bioinformatic tools are being developed for biomarker discovery and validation. Understanding basic concepts and application of such technology by investigators in the field may accelerate the clinical application of protein biomarkers in disease management.

## Introduction

Cancer is a prevalent and serious public health problem not only in the United States and also worldwide. It is a major source of morbidity, and the second leading cause of death in the American population. A total of 1,444,920 new cancer cases and 559,650 deaths are projected in the United States in 2007 (Jemal et al. 2007). When deaths are aggregated by age, cancer has surpassed heart disease as the leading cause of death in individuals 85 years age or younger since 1999 (Jemal et al. 2007).

Before cancer prevention becomes a reality, early detection and better treatment remain the hope for all cancer patients. It is easy to understand that the test for early detection of cancer is best done in blood or other types of body fluids; however, cancer classification and management are frequently accomplished by using tissues, as it truly represent the source for a much more reliable discrimination between different types and subtypes of cancer. Protein biomarkers in blood or tissues may therefore play an important role in cancer detection, monitoring and treatment.

Proteomics is a rapidly advancing field brought about in large part because of the recent developments in mass spectrometry and associated technologies. These developments allow for the fast and reliable detection, identification and relative quantitation of proteins. The measurements can be made from the solid and solution states, and sometimes from complex mixtures. The amount of protein required for these measurements has been continuously reducing, and today many laboratories have demonstrated the ability to obtain results from samples with protein concentration as low as 5 femtomoles (about 3 × 10^−10^ grams of albumin, for example). Emerging from this work is a new goal in clinical research to use these techniques to discover and identify proteins that are specific to, and diagnostic for, a given disease. If successful, these developments will ultimately allow for early detection and possibly individualized therapy for the disease. The object of this review is to describe the basic concepts, tools, limitations and progress in this rapidly advancing field. The obvious potential of protein biomarker screening for cancer diagnosis has yet to be fully realized. The clinical application of proteomics to cancer management will not be successful until well controlled investigations with standardized methods of proven accuracy for profiling nanogram level proteins in complex mixture of biological specimens.

## Why protein biomakers?

There are many reasons why proteins are important or even to be better biomarker than other molecules such as DNA or RNA. Firstly, proteins are much more diverse than nucleic acids and therefore carry more information. Through alternative splicing and over 100 unique post-translational modifications (PTMs) including covalent modification by attachment of specific functionalities (acetylation, phosphorylation, etc), as well as post-translational trimming and cleavage of proteins by site-specific proteases, tens and possibly hundreds of distinct protein species can result from expression of a single gene. Moreover, many cellular functions are not revealed at the level of the nucleic acids, but are manifested by the PTMs, as well as cleavage of proteins by site-specific proteases. With a limited number of human genes (~20,000) and many more proteins (10^5^–10^6^) (Stein 2004; Pennisi 2003), the latter are likely to more closely reflect the complex nature of cellullar biology and host physiology. Secondly, fluctuating levels of an mRNA transcript make the correlation with the amount of protein synthesized inconsistent in many cases. For example, when the same cells or tumors were examined by both cDNA arrays and proteomic analysis, the concordance rates were low between the mRNA transcript profiles and corresponding protein expressional patterns (Anderson and Seilhamer, 1997; Alaiya et al. 2000a; Chen et al. 2003; Nishizuka et al. 2003). Therefore studies limited to the DNA or mRNA analysis may reflect only a partial assessment of cellular function. Documenting proteins and their interactions in cancer cells represents a complementary and much more comprehensive approach to understanding the cellular changes caused by disease processes.

## Definitions

A “*biomarker*” is defined as a measurable analyte that correlates with a specific phenotype, such as a normal biological condition, a pathological process, or a pharmacological response to a therapeutic intervention (Group, 2001). A biomarker is unique in that it distinguishes and discriminates between comparative biological conditions such as cancer and non-cancer. “*Cancer proteomics*” is defined as molecular profiling of cancer-associated proteins approached by analyzing the global protein expression patterns of tumor cells or extracellular fluids such as serum from cancer patients (Alaiya et al. 2000b). To date there have been many candidate proteomic biomarkers suggested for various types of cancer. However, none has yet met the stringent requirements for clinical use, such as reproducibility, specificity and sensitivity. A true biomarker should be confirmable across laboratories and technology platforms (Aebersold et al. 2005), in its ability of predicting the clinical state. Today the greatest need in cancer proteomics research lies in improvement in the methodology and technology for the recognition and identification of reliable protein biomarkers specific for the disease or biology of the disease.

Historically, biomarker discovery was dominated by targeted approaches, in which candidates derived from biological knowledge were selectively evaluated for their correlations with clinical conditions (Gillette et al. 2005). However, in the past two decades, the advances in new mass spectrometric ionization techniques for discovering macromolecules has revolutionized biomarker research (Karas and Hillenkamp, 1988). Now for the first time it is possible to display complex protein profiles in convenient one- and two-dimensional formats, and at the same time identify the proteins. More and more studies are employing mass spectrometry technology to search for novel cancer biomarkers. Reflecting this trend, in March 2007, a MedLine search for the words “cancer biomarker” listed in 119,912 publications, while a similar simultaneous search that included the word “protein” resulted in 96,598 articles. A selection of published representative studies on cancer biomarkers using mass spectrometry technique are summarized in Table 1. However, as reported by Alaiya et al. (2005), the lack of proteomics markers for cancer stands in sharp contrast to the significant progress that has been made in the discovery of gene-based biomarkers during the last 20 years. This disparity also reflects the increased difficulty when working with the proteome as opposed to the genome. Nevertheless, the potential offered by proteomic technology in biomarker discovery draws research support from both government funding agencies and private sector.

A protein biomarker detected by mass spectrometry can be a single entity (with protein identification, therefore identity-based) or a suite of entities (protein signatures, therefore pattern-based). For the biomarker to be clinically useful, the abundance or change in abundance of specific proteins must reflect some aspects of normal physiology/biochemistry or a disease process. Combined liquid chromatography-mass spectrometry (LCMS) is the technique to which most cancer proteomic research is now gravitating. Because of the direct on-line link between chromatography and mass spectrometry, this technique can more easily accommodate complex biological samples such as serum and plasma. In contrast, the enthusiasm which originally greeted other techniques that do not use direct on-line chromatography, such as surface enhanced laser desorption mass spectrometry (SELDI), has now waned. This has occurred as the need for chromatography when dealing with complex samples, such as serum and plasma, has become obvious. In this context then, as defined here, a biomarker may be a *peak or peaks* detected on a chromatogram, a *signal or signals* in a mass spectrum, or a *feature or features* in a three dimensional representation of peak retention times, signal mass to charge (m/z) ratios and intensities. These are three different representations of LCMS data that are routinely acquired in proteomic analyses.

It has been pointed out that the utility of a protein biomarker for disease diagnosis or detection does not necessarily require knowledge of the identity of the protein or proteins involved (Petricoin et al. 2002; Villanueva et al. 2005), so long as the pattern of peaks, signals or features are sufficiently reproducible to reflect the present disease. Strictly speaking, this could be the case. However, without knowing the identity of the biomarker proteins, information related to the underlying disease process will not be forthcoming. Furthermore, the identity of the protein or proteins involved is necessary for independent validation of the biomarker by different technologies, and for the future development of faster, cheaper and more reliable assays (which probably will not rely on chromatography or mass spectrometry). Consequently, disease-based protein biomarker research involves both recognition of the biomarker signature and identification of the proteins involved.

## A dilemma—low- and high-abundance proteins

The components of complex samples (proteomes) are typically present in a wide concentration range. The components of human plasma and serum for example extend across 10–12 orders of magnitude in concentration (Anderson and Anderson 2002). This presents an analytical dilemma because no one technique has a dynamic range that can accommodate such samples. In the case of cancer biomarker screening, while the results of some investigations (Li et al. 2005, Yu et al. 2004) suggest that high abundance proteins such as Complement C3a (desArg) (a 77-amino acid protein) and a C-terminal-truncated form of C3a may be useful for diagnosing breast cancer, and β-globin for colorectal cancer, the low specificity of these tests limits their clinical utility. At the same time, the most clinically useful cancer biomarkers are low-abundance molecules; like prostate specific antigen (PSA) (Wang et al. 1979; Stamey et al. 1987), members of the mucin family of proteins (for example, CA 15.3), carcinoembryonic antigen (CEA) and cytokeratins (i.e. TPA, TPS and Cyfra 21.1; Seregni 2004) being examples. Interestingly, all these established cancer biomarkers are glycoproteins. In addition, the alteration in protein glycosylation which occurs through varying the heterogeneity of glycosylation sites or changing glycan structure of proteins on the cell surface and in body fluids have been shown to correlate with the development of cancer and other disease states (Durand and Seta, 2000). Therefore, the technology for screening protein biomarkers in body fluids must confront the large dynamic range of the concentration of the molecules of interest. Thus to accommodate both low- and high-abundance molecules, there is a need for fractionation of complex samples. While undesirable because it increases the workload and introduces additional error in the measurements, this seems unavoidable. One current research focus is how best to fractionate complex proteomes, and many different strategies are now being compared.

## Application of proteomic technologies to cancer

### Early detection of cancer

It is well known that early detection of cancer can lower the mortality rate. Cancers diagnosed at an early stage are more likely to be cured by conventional therapy. For many cancers, 5 and even 10-year survival may approach or even exceed 90% if treated early, and only 10% or less when found late (Etzioni et al. 2003). The Pap smear test (named after the inventor Georgios Papanicolaou) for early detection of pre-neoplastic cervical cancer (Shingleton et al. 1995), and colonoscopy for early detection of colon cancer (Winawer et al. 1995; Winawer, 2001), are good examples of how early disease detection can significantly improve cancer outcome. Not surprisingly, the focus therefore in biomarker screening is to identify proteins that can aid in early detection and diagnosis of the disease.

Similar to other cancers, the early detection of breast cancer by serum biomarkers currently remains a research quest. The circulating breast cancer markers now used in clinical practice (CA 15.3, CEA and cytokeratins) are useless in early detection since they are all associated with a large tumor burden at late stage of the disease (Seregni et al. 2004). It is estimated that a minimum number of 10^9^ tumor cells are required for breast cancer detection by the current methods such as mammography or clinical breast examination (Dummin et al. 2006). Therefore the challenge is to develop techniques that can detect specific disease-related changes from cells fewer than 10^9^ cells or a total mass equivalent to milligrams of tissues. Fortunately, the field of cancer biomarker screening is evolving rapidly, and recent developments in the technological, biological and statistical realms show promise for uncovering markers that meet this goal.

In 2002, Petricoin et al. made a sensational report of mass spectrometry/proteomics-based methods for the detection of ovarian cancer. In this work SELDI mass spectrometry was used to profile serum proteins, the pattern of which could be used to separate cancer from non-cancer patients. One claim was that the technology could be used for early detection of ovarian cancer. In the study, a preliminary “training” data set, consisting of spectra derived from the analysis of serum from 50 unaffected women, and 50 patients with ovarian cancer, was analyzed by an interactive pattern-recognition algorithm. A pattern was identified that discriminated cancer from non-cancer. The pattern was then used to classify an independent set of spectra obtained from 116 masked serum samples collected from 50 women with ovarian cancer, and 66 unaffected women. The pattern identified in the training set successfully segregated cancer from non-cancer in the second set of samples. The validation set correctly identified all 50 ovarian cancer cases, including all 18 stage I cases. Of 66 cases of non-malignant disease, 63 were classified correctly, giving a sensitivity of 100% (95% confidence interval (CI) 93–100%), a specificity of 95% (CI of 87–99%), and a high positive predictive value of 94% (CI of 84–99%).

Since publication of this report, there have been several attempts to repeat, confirm and extend these findings to other types of cancer. These newer investigations have used similar proteomic profiling approaches with body fluids from patients with various types of cancer (Adam et al. 2002; Li et al. 2002; Borozdenkova et al. 2004; Ebert et al. 2004, Li et al. 2005; Mendrinos et al. 2005; Villanueva et al. 2005). Unique biomarker patterns have been reported for early detection of ovary, prostate, breast, and thyroid cancer. The enthusiasm led to the postulation that a new era in cancer diagnostics had come, in which serum proteomic profiling would meet the goal of early cancer detection.

However, several methodological and bioinformatic artifacts and biases have been identified, which challenged the validity of the published results (Baggerly et al. 2005; Diamandis and van der Merwe, 2005). While it appears that all have high diagnostic sensitivities and specificities, few of the reported biomarkers have adequate reproducibility (Diamandis, 2002). In retrospect it appears that the limitations of these initial efforts to identify reliable diagnostic cancer biomarkers resulted from differences in methodology among the investigators.

Like almost every newly emerged technology, through these early efforts including errors and fails in searching disease associated biomarkers, much has been learned to pave the way for true biomarker discovery. The expectation to the new technique frequently exceeds the capacity at the moment. On the other hand, the unfulfilled expectations or the aware limitations of the technique are the forces to drive the research forward.

In the foreseeable future, the field will be continuously challenged by the heterogeneous nature of the disease, the specimen and the technology. It is quite often the case that the heterogenesis of patients, such as gender, age, genetics, ethnicity, body mass, medication, the presence of other conditions and diseases, emotional stress, menopausal status, and dietary/nutritional preferences, as well as sample preparation, may all introduce artifacts. Also, given the small quantity of the human specimens and the limited availability of quality samples, and the extraordinary cost of proteomics technologies, many studies are based on small numbers of samples or even pooled samples. Thus, with small sample numbers, it is reasonably expected that the overlap of findings among different groups would be low. As progress being made rapidly, the findings would eventually grow together. To make the matter even more complex, the mass spectrometry platforms and methodology used drastically could be different among research groups. Even if the instruments are same, they could be operated in very different modes for which those details are not disclosed in publications. Thus, the MS methodologies used are as heterogenous as cancer disease itself. Taking together, it becomes clear that consistent findings amongst laboratories can only accomplished after.

Another fundamental problem, as already mentioned, lies in the enormous number and 10^9^concentration range of proteins in tissues and body fluids and the complexity of these fluids. Compared with MS techniques, current cancer markers identified by immunological techniques are at subnanogram levels, and have no cross-reactivity in the presence of a huge excess of other unrelated proteins. Immunological techniques avoid the need for removal of high abundance proteins before nanoscale detection of the target analyte. However, compared with immunological techniques, the unique promise of proteomics results from its potential to simultaneously resolve and compare thousands of proteins for qualitative and quantitative differences prior to identification by mass spectrometry. This technology will become a powerful diagnostic tool once methods are developed to first separate high, medium, and low abundance proteins before proteomics analysis of each of these three subsets using the more than 1,000-fold dynamic range capability of mass spectrometry.

### Proteomics in cancer prognostics and monitoring response to therapy

Increased serum levels of proteins like CEA and PSA are used to detect re-growth of some common malignancies after conventional therapy including surgery, radiation, and chemotherapy. Progression of nearly all tumors results from induction of inflammatory cytokines, growth factors, angiogenic peptides like VEGF, and release of high abundance coagulation proteins in the blood that stimulate cancer cell proliferation and metastasis. Many of these cancer-promoting peptides are transported by plasma lipoproteins that have been identified recently by a combination of proteomic approaches including 1-DE and 2-DE MALDI-TOF, isotope-coded affinity tag and Western blot analysis (Rezaee et al. 2006). This provides an unprecedented opportunity to apply current proteomics technology to cancer prognosis by monitoring serum and plasma protein levels after primary or adjuvant therapy.

In addition to the effort investigated in early detection, chemotherapy has been widely used in the treatment of various cancers for reducing cancer mortality. However, unpredictable effectiveness and treatment toxicity continue to plague the chemotherapy. The novel treatments that are focused on specific targets in the signal transduction and/or metabolic pathways are extremely effective, and have less treatment associated toxicity. Herceptin^®^ (trastuzumab), a humanized monoclonal antibody which targets tumors over-expressing the Her-2/neu receptor protein (Baselga et al. 2004; Ross et al. 2004a, b), and Gleevec^®^ (imatinib mesylate), targeting cancers with bcr-abl gene translocation in chronic myeloid leukemia (Druker, 2004), are two of the best examples. The success of Herceptin and Gleevec has stimulated the development of pathway specific treatments for other types of cancer. Protein biomarker screening offers potential for subtyping cancers according to their unique protein profiles. Such signatures could be used to guide tailored treatment, avoid unnecessary toxicity, and reduce cost because of the selective nature of the treatment. Signatures with biological significance may not only improve cancer sub-classification but also lead to the development of novel treatments (Hunt and Keyomarsi, 2005).

## Clinical proteomic approaches and platforms

### Specimens for protein profiling of cancer

Many types of biological specimens have been used in cancer proteomic research, such as cell lines, tumor tissue, serum, plasma, urine, salvia and nipple aspirate fluid (NAF) for breast cancer (He et al. 2007; Shau et al. 2003). Tumor tissue may be an ideal source to study cancer proteomic signatures. Unfortunately tumor tissue can be difficult to obtain and to preserve for proteomic analysis. Serum or plasma on the other hand has been most commonly used for biomarker research because sufficient amounts of these samples are easily attainable by relatively non-invasive means, and frequently contain tumor markers, albeit maybe in low abundance. It is known that not only do tumors leak or secrete proteins into circulation, but also the surrounding stroma releases proteases and other mediators of tissue injury in response to the nearby tumor growth. The currently used tumor markers such as PSA, CA125, CEA, and alpha-fetoprotein (AFP), are all examples of useful low abundance circulating cancer biomarkers that probably arise from the tumor and/or the surrounding stroma (Bast et al. 2001; Dhanasekaran et al. 2001).

However, serum/plasma profiling is not without its challenges. As already alluded to, one major obstacle is the wide concentration range of the candidate markers that exceeds the dynamic range of any single analytical method or instrument (Domon and Aebersold, 2006). For example, it has been estimated that the concentration range of various serum proteins exceeds 10 orders of magnitude (Anderson and Anderson, 2002). Although this represents a daunting hurdle, recent technological developments have increased the dynamic range of analytical instrumentation, and new methodological developments have reduced the threshold for protein detection to sub-nanogram levels (Domon and Aebersold, 2006).

### Fractionation of plasma and serum proteome

It is generally accepted that any method for detection of disease biomarkers in complex proteomes such as plasma and serum will require fractionation to reduce the complexity of the sample. This is considered essential even after the major abundant proteins, such as albumin, hemoglobin, immunoglobulin, transferrin, complement, haptoglobin and others, have been depleted from the sample (Echan et al. 2005). A reflection of this trend can be seen in a number of methods already reported for accomplishing this purpose. Numerous strategies have been developed including ion exchange based separations using the combination of strong cation exchange chromatography with reversed phase separations such as the multidimensional protein identification technology (MUDPIT) developed by Yates and co-workers (Washburn et al. 2001), molecular weight based filtration (Hu et al. 2005), hydrophobicity based separation such as the use of reverse phase separations (Wang and Hanash, 2005), the capture of cysteine-containing peptides with biotinylated thiol reagents (Schrimpf et al. 2005), immobilizing of phosphorylated peptides by metal affinity chromatography (Corthals et al. 2005), dendrimer conjugation chemistry (Tao et al. 2005), glycopeptide capture (Zhang H. et al. 2005), activity-based protein profiling (ABPP) (Speers and Cravatt, 2004), fluorophosphonate (FP)-based ABPP (Jessani et al. 2005) targeting serine hydrolase, monolithic columns and Beckman’s PF2D fractionation instrument (Ratnayake et al. 2000). Also, there has been extensive work on microseparations for froteomic studies by Cheng Lee’s team (Li et al. 2003) and Smith’s group (Smith, 2006). Finally, Wall et al. (2000) developed a unique two-dimensional all liquid-phase method combined with MS for protein profile analysis. With this method, proteins are fractionated by p*I* using isoelectric focusing (IEF) in the Rotofor cell and then separated by hydrophobicity using reverse phase-HPLC in the second dimension. All these separation or depletion procedures may help to remove interfering proteins and allow detection of biomarkers with lower concentrations for the analysis of cancer proteome.

### Methodology and proteomic platforms

The basic principles of proteomics methods currently used in the application of cancer research including two-dimensional gel electrophoresis (2DE) and mass spectrometry (MS). Although 2DE is still a currently used tool for proteomic analysis, it has disadvantages regarding to its throughput, reproducibility, sensitivity and limited range of MW (<200 kDa) and p*I* (between 4 and 10), which limit the use of this method (Jenkins and Pennington, 2001). This review will focus on proteomic techniques based on mass spectrometry for cancer biomarker research.

Since the discovery of new mass spectrometric ionization techniques for macromolecules almost 20 years ago (Karas and Hillenkamp, 1988), many MS instruments have been developed and used in cancer biomarker research, for example, MALDI-TOF MS, SELDI-TOF MS, LCMSMS and Qstar.

The term matrix-assisted laser desorption ionization (MALDI) was coined in 1985 by Franz Hillenkamp, Michael Karas and their colleagues (Karas et al. 1985). A matrix is used to protect biomolecules from being destroyed by direct laser beam. Using laser and matrix combination, ionization of large biomolecules is possible (Karas and Hillenkamp, 1988). Further improvements were realized through the use of a 355 nm laser and the cinnamic acid derivatives ferulic acid, caffeic acid and sinapinic acid as the matrix (Beavis et al. 1989). Today, MALDI-TOF MS became a popular and versatile method to analyze macromolecules from biological origin. In combination with 1DE and 2DE separation, MALDI-TOF is used to discover disease markers. For example, peptide mass fingerprint (PMF) is a protein identification method based on the specificity of a mass spectrum of the peptide mixture resulting from the digestion of a protein by an enzyme (e.g. trypsin). After proteolysis with a specific protease, proteins of different amino acid sequence produce a series of peptides masses, which can be detected by MALDI. The spectrum of identified peptide masses is unique for a specific protein and is known as a mass fingerprint (Marvin et al. 2003). Searching the selected masses from the fingerprint against databases of known protein sequences (e.g. SwissProt-TrEMBL) enables the identification of most proteins.

SELDI technology was developed by Hutchens at Baylor College of Medicine in 1993 (Hutchens and Yip 1993). The technology was commercialized by Ciphergen Biosystems Inc. in 1997 as the ProteinChip system. In this method, proteins are captured directly on a chemically derivatized MALDI plate. SELDI-TOF MS is similar to MALDI-TOF. They differ in how the matrix, or energy-absorbing molecule, is mixed with the protein sample. In MALDI, a protein or peptide sample is mixed with the matrix molecule in solution. Small amounts of the mixture are “spotted” on a surface and allowed to dry. The peptide sample and matrix co-crystallizes as the solvent evaporates. In SELDI the protein mixture is spotted on a surface modified with some chemical functionality. Some proteins in the sample bind to the surface, while the others are removed by washing. After washing the spotted sample, matrix is applied to the surface and allowed to crystallize with the sample peptides. Binding to the SELDI surface acts as a chromatography step and the subset of proteins that bind to the surface are easier to analyze. Common surfaces include CM10 (weak-cation exchanger), H50 (hydrophobic surface, similar to C6-C12), IMAC30 (metal-binding surface), and Q10 (strong anion exchanger). Surfaces can also be functionalized with antibodies, other proteins, or DNA.

SELDI allows the discrimination of peptides based on mass over charge ratio and provides a semi-quantitative evaluation, but cannot identify these peptides (Fung et al. 2001), which is the major disadvantage of this technology. So each protein of interest has to be purified or enriched for identification purpose. The application of SELDI led to many exciting results (Petricoin et al. 2002), although results were not always reproducible (Editorial, 2004; Sorace and Zhan, 2003; Baggerly et al. 2004). In Table 1, a list of several representative SELDI publications in cancer biomarker research was posted to reflect the historical interest in this technology during the time period. While the enthusiasm in traditional SELDI declining, MALDI-TOF MS which is a particle-counting method that responds to molar abundance, still represent a useful tool for surveying small proteins and peptides. It is complementary to techniques such as electrophoresis and HPLC, which have an advantage for detecting larger molecules (Hortin, 2006).

**Figure 1:**
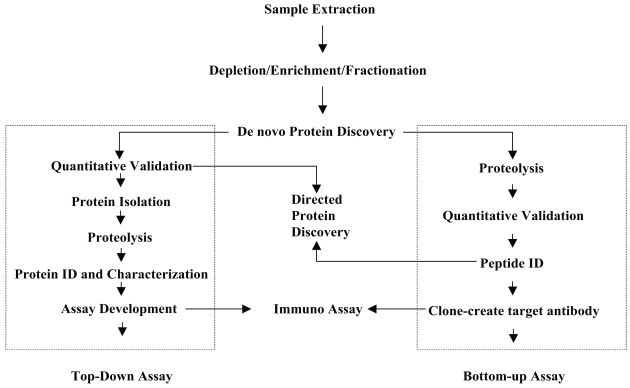
Proteomic biomarker discovery: integration of Top-Down and Bottom-Up approaches.

The methods used to explore cancer biomarkers will be discussed to facilitate the understanding of different techniques involved in different strategies. This can be simply categorized into two groups: the “bottom up” (peptide level) and “top down” (intact protein level) approaches (Figure 1, from Dr. Weinberger of GenNext Technologies^™^ Inc. with permission). The “bottom up” approach involves protein digestion followed by mass measurement of the resulting peptides and subsequent determination of partial sequence of the peptides. This data set is then compared against a data set composed of theoretical peptides, their masses and sequences for prediction of the identity of the peptide or protein in question. The result of this comparison is a report of the matches between the measured peptides and the theoretical peptides, usually listed in order of decreasing strength of the match. The top down approach first involves mass measurement of the intact protein followed by attempts to identify the protein on the basis of this value and the measured molecular weights and sequences of the peptides derived from each digested protein. Careful and thorough examination of plasma/serum proteomes for disease biomarkers identification should embrace both approaches because each has their own advantages and disadvantages. For example, the bottom-up approach has been successfully adapted to high throughput screening of complex proteomes but it lacks the molecular weight information of the intact protein and is less effective in recognizing and identifying the presence of PTMs (Bogdanov and Smith, 2005).

Few of the known protein biomarkers of cancer are exclusive for a specific malignancy, and most are also found in non-malignant conditions. Current cancer biomarkers are associated with an abnormal temporal, quantitative or conformational presentations (i.e. in incorrectly spliced or post-translationally modified forms), or combinations thereof. Although some of these associations are mere reflections of abnormalities downstream of the real pathogenesis of the disease, it does not detract from their value as markers for cancer detection or stratification. Consequently, the most useful cancer biomarkers are likely to be a suite of proteins that change in relative abundance during the disease process and during treatment. The implication of this is that the technology to detect these biomarkers must be both qualitative and sufficiently quantitative to record subtle changes in the plasma/serum proteome in the face of a large number of other proteins or after depletion procedures.

To this end, significant investments have already been made in the exploration of the serum/plasma proteome to diagnose disease (Conrads et al. 2005, 2006). Electrospray (Whitehouse et al. 1985; Mora et al. 2000; Fenn, 2003) and laser ionization mass spectrometry have become important in this process as the most robust methods for ionizing a wide range of proteins and peptides prior to their mass spectrometric analysis. Several other important techniques have been used effectively to probe complex proteomes. Several important techniques are useful in this effort. The MUDPIT technology (Washburn et al. 2001) has already been described for the successful fractionation of complex proteomes. Tryptic digestion of the mixture is followed by two or more steps of sequential liquid chromatography coupled with ESI mass spectrometry. Isotope coded affinity tags (ICAT, Gygi et al. 1999) and the newer, more successful technique of isobaric tags for relative and absolute quantification (iTRAQ, Zhang Y. et al. 2005) both use stable isotope- labeled reagents for relatively quantitative comparisons of the proteomes of two or more samples. These strategies are based on the reaction of protein mixtures using reagents with specificity toward certain functional groups such as the free sulfhydryl groups on cysteine residues. The reagents contain components with different molecular weights and an affinity tag. Samples are separately reacted using the reagents with different molecular weights. The samples are then mixed. The same peptides present in multiple samples differ in molecular weight and can be distinguished by MS, and their relative signal intensities accurately reflect their relative abundances in the original samples. Extrapolation of such approaches for a comparison of relatively large numbers of samples, such as will be collected in the course of clinical trials, is an issue with which the field has yet to grapple. Also iTRAQ technique labels all peptides in a sample making affinity separations unnecessary. Similarly, iTRAQ reagents are all isobaric, the difference in mass only becomes apparent due to the “reporter ion” in the MS/MS dimension. Finally, Wall et al. (2000) developed a unique two-dimensional all liquid-phase method combined with MS for protein profile analysis. With this method, proteins are fractionated by p*I* using isoelectric focusing (IEF) in the Rotofor cell and then separated by hydrophobicity using reverse phase-HPLC in the second dimension.

## Reverse phase protein microarray (RPMA) in clinical phosphoproteomic profiling

Besides mass spectrometry technology, protein microarray technology has also been widely used in proteomic studies. Protein microarrays can be divided into two broad categories: forward phase microarrays (FPMA) and reverse phase microarrays (RPMA). In the FPMA format, the analyte is captured from solution using a “bait” molecule immobilized on the array substrate. In contrast, in the RPMA format, the analyte is immobilized directly on the array substrate before being probed with an analyte-specific ligand, usually an antibody (Liotta et al. 2003; Templin et al. 2002; Mundinger et al. 2006).

RPMA technology, first introduced in 2001, is well suited to clinical proteomic research of oncology. One of the advantages of RPMA is to measure multiple analytes simultaneously from relatively smaller numbers of cells than required by mass spectrometry tools (Mundinger et al. 2006). Another advantage is to study the phosphorylation and dephosphorylation events mediated by protein kinases, which are critical in transduction networks and their aberrancies in cancer constitutes an exciting frontier in oncology (Pawson, 2002, Mundinger et al. 2006). Moreover, this method is highly sensitive with detection capabilities of 50 fg/l, or 1,000 to 5,000 molecules per spot (Liotta et al. 2003; Paweletz et al. 2001; Geho et al. 2005). However, the limitations of this analytical tool are that the quality of the arrays depends, as in all immunoassays, on the performance of the primary antibody used. The endogenous biotin, immunoglobins, peroxidases, alkaline phosphatases, or fluorescent proteins, contained in biological samples, have the potential to interfere with amplification methods currently used for array detection (Mundinger et al. 2006). And finally since RPMA is dependent on antibodies, it cannot identify novel protein species, although it may identify novel network interactions among previously characterized proteins (Jones et al. 2006).

To date, several studies have utilized RPMA in the analysis of cancer signaling pathway alterations with clinical prostate (Paweletz et al. 2001; Grubb et al. 2003; Herrmann et al. 2003), ovarian (Wulfkuhle et al. 2003; Sheehan et al. 2005), colorectal (Belluco et al. 2005), breast cancer (Zhang DH et al. 2005a, b; Cowherd et al. 2004, Wulfkuhle et al. 2002; Kersting et al. 2004) and lymphoma (Zha et al. 2004; Gulmann et al. 2005) specimens using RPMA. In these studies, pro-survival, pro-mitogenic, and cell-cycle regulatory proteins, including phospho-Akt, PI3K, ERK, MAPK, PKCα, p38, STAT1, GSK3-β, cytochrome c oxidase, c-erbB2, and c-erbB1, phospho-PTEN, EGFR, have been studied.

## Advantages and pitfalls of current proteomic technology

Proteomic technology enables high throughput analysis of protein biomarkers, and therefore provides an opportunity to identify and evaluate all potential protein biomarkers for early detection and to predict various tumor behaviors, including response to chemotherapy. This approach is gaining popularity among cancer researchers in their quest for cancer biomarkers with high diagnostic, prognostic and predictive accuracy. However, despite initial excitement, skepticism about the methodology and the lack of concordance of results among labs and even within the same laboratories has eroded confidence in this technology (Diamandis 2004a, 2004b; Baggerly et al. 2004; Check, 2004; Villanueva and Tempst, 2004; Ransohoff, 2005). The simple fact that different research groups have found different discriminatory markers when analyzing similar samples suggests that the methodology needs to be standardized to improve comparability, reproducibility and reliability of the findings.

Apart from reproducibility and reliability of the mass spectrometric technology, other factors may also complicate the analytical effort. For example, sample collection, storage, and processing procedures can produce proteomic artifacts that could overshadow those representing of cancer (Villanueva et al. 2005; Banks et al. 2005; Karsan et al. 2005). Also the protein turnover rates in serum may be affected by liver and kidney function of each individual. It is known that cancer-associated biomarkers are also released by conditions other than cancer, which lowers specificity of the approach. Therefore, extreme sensitivity may be required because crucially important proteins often exist at low concentration in a particular type of cancer at its early stage.

Detection of proteins released from tumor cells into the circulation is a challenging task, so prudent choices need to be made when selecting the proper technology and strategy. Depending on the experiment and the analyte, the sensitivity of proteomic LCMS experiments is now down to the 50 fmol to 10 attomol of analyte-injected range. This translates to 5 ngm to 1 pgm of a 10 kDa protein. If the analyte were recovered from 1 ml of specimen, this limit of detection (LOD) is equivalent to or below the concentrations of most known circulating tumor markers, for example PSA (Maattanen et al. 2001). Therefore, mass spectrometric detection is now at the level of mid-range circulating biomarkers. Although further improvements are needed before the low-range circulating biomarkers become accessible, the improvements in mass spectrometric LOD’s in the last few years have been remarkable. Nevertheless, discriminatory peaks may include acute-phase reactants (i.e. molecules whose serum concentrations are increased with acute or chronic inflammatory conditions) or other proteins or protein fragments that are released into the circulation by large organs, such as the liver, in response to the presence of a tumor or cancer, but not from cancer itself. Such epiphenomena can be mimicked by condition other than cancer including infection, inflammation, or malnutrition (Diamandis 2003a, 2003b, 2004a, 2004b).

In addition, numerous patient and environment-related variables such as gender, age, genetics, ethnicity, body mass, medication, the presence of other conditions and diseases, psychological distress, menopausal status, and dietary/nutritional preferences, may all introduce artifacts, and the effect of these factors on the serum/plasma proteome have yet to be systematically investigated.

In summary, designing a protein/peptide profiling study must be rigorous to control for all important variables. Furthermore, standardized and optimized methodology is essential for achieving accurate measurement and meaningful analysis. This includes all involved steps extending from experimental design, specimen collection, storage and handling, throughout all methods used in the analytical chemistry and MS signal processing. Proper bioinformatics including analytical tools, data storage and sharing are required for data mining and validation.

## Figures and Tables

**Table 1:** Summary of selected MS-based cancer biomarker studies.

Cancer type	Specimen	Diagnostic sensitivity and specificity or protein ID	MS technology	Reference
Breast	Serum	93%; 91% Complement component C_3adesArg_ and its truncated form	SELDI-TOF, Tandem mass peptide sequencing	Li et al. 2002, 2005
Breast	Ductal lavage	75%	SELDI-TOF	Mendrinos et al. 2005
Breast	Nipple aspirate fluid	Hb-β chain	SELDI-TOF	Sauter et al. 2005
Breast	Serum	High molecular weight kininogen, apolipoprotein A-II.	SELDI-TOF	Heike et al. 2005
Breast	Serum	94%; 81.3%	2DE, LCMSMS	Ru et al. 2006
Breast	Nipple aspirate fluid	Vitamin D binding protein et al..	ICAT MSMS	Pawlik et al. 2006
Breast	Cell line	14-3-3 sigma	MALDI	Liu et al. 2006
Breast	Tissue	Thymosin alpha-1	HPLC and MALDI	Traub et al. 2006
Breast	Nipple aspirate fluid	8 markers with m/z – 5061, 5994, 6001, 10207, 13070, 13436, 13447, 57075	SELDI-TOF MS	He et al. 2007
Breast	Tissue	>1000 protein identified	NanoLC MS	Umar et al. 2007
Ovarian	Serum	100%; 95%	SELDI-TOF	Petricoin et al. 2002
Ovarian	Serum	73–96%; 83–95%	SELDI-TOF	Kozak et al. 2003
Ovarian	Serum	Haptoglobin-alpha subunit	SELDI-TOF	Ye et al. 2003
Ovarian	Serum	Apolipoprotein A1, truncated form of transthyretin, fragment of inter-alpha trypsin inhibitor heavy chain H4. 74%; 97%	SELDI-TOF	Zhang Z et al. 2004
Ovarian	Serum	Transthyretin and its fragment, beta-hemoglobin, apoAI, and transferrin	Micro-LCMSMS	Kozak et al. 2005
Ovarian	Serum	Hemoglobin-alpha and -beta	SELDI-TOF	Woong-Shick et al. 2005
Ovarian	Plasma	94%	SELDI-TOF	Rai et al. 2002
Ovarian	Plasma	Amyloid A1 and its truncated form	SELDI-TOF	Moshkovskii et al. 2005
Ovarian	Plasma	90–96.3%; 100%	SELDI-TOF	Lin et al. 2006
Ovarian	Plasma	fibrinopeptide-A	2DE, MS	Ogata et al. 2006
Endometrial	Tissue	95%, chaperonin 10, a-1-antitrypsin	LCMSMS, Q-STAR	Desouza et al. 2007
Colorectal	Serum	95.2%, 90.0%	MALDI-TOF	De Noo et al. 2006
Colorectal	Cell line	Prothymosin α	SELDI-TOF	Shiwa et al. 2003
Colorectal	Serum	N-terminal fragment of albumin, apoC-I, apoA-I	SELDI-TOF	Engwegen et al. 2006
Colorectal	Serum	β-defensins	SELDI-TOF	Melle et al. 2005
Colorectal	Tissue, serum	β-defensins	SELDI-TOF	Albrethsen et al. 2005
Colorectal	Tissue	Hydrophobic proteins (vimentin)	2DE, MALDI-TOF/TOF, ESI–MSMS	Alvarez-Chaver et al. 2006
Prostate	Serum	Zn-alpha2 glycoprotein (ZAG)	LCMSMS	Bondar et al. 2007

## Abbreviations

2DE: two-dimensional gel electrophoresis
ABPP: activity-based protein profiling
CEA: carcinoembryonic antigen
CI: confidence interval
ESI: electrospray ionization
FP: fluorophosphonate
HPLC: high performance liquid chromatography
ICAT: isotope coded affinity tags
IEF: isoelectric focusing
iTRAQ: isobaric tags for relative and absolute quantification
LCMS: combined liquid chromatography-mass spectrometry
LCMSMS: liquid chromatography tandem mass spectrometry
LOD: limit of detection
m/z: mass to charge ratio
MALDI: matrix-assisted laser desorption ionization
MS: mass spectrometry
MUDPIT: multidimensional protein identification technology
NAF: nipple aspirate fluid
PMF: peptide mass fingerprinting
PSA: prostate specific antigen
PTMs: post-translational modifications
RPMA: reverse phase protein microarray
SELDI: surface enhanced laser desorption ionization
TOF: time-of-flight
